# Cardiac Troponin T in Neonates from Normal and Gestational Diabetes Mellitus Pregnancy

**DOI:** 10.1155/2022/6171687

**Published:** 2022-10-18

**Authors:** Joanna Kłaczewska, Ryszard Tomasiuk

**Affiliations:** ^1^Józef Polikarp Brudzinski Children's Clinical Hospital in Warsaw, 63A Żwirki i Wigury St., 02-91 Warsaw, Poland; ^2^Kazimierz Pulaski University of Technology and Humanities Radom, Faculty of Medical Sciences and Health Sciences, Radom, Poland

## Abstract

This study is aimed at testing the hypothesis that serum analysis of high-sensitivity troponin T in neonates may serve as a diagnostic tool to predict the risk of gestational diabetes mellitus (GDM). 86 newborns were studied and stratified into two groups: healthy group; newborns with body weight ≥ 10th percentile, born in good condition (APG 8-10pts) of pregnancy not complicated by diabetes, and the GDM group; neonates born to mothers with type 1 or type 2 diabetes. *Results*. The study revealed minimal troponin levels in GDM, and healthy groups equal to 0.02 ng/mL and 0.028 ng/mL, respectively. The GDM group is defined by an interquartile range of hs-TnT higher than the healthy group. This study confirms previously reported upper levels of troponin in healthy children. There are possible health problems that can appear during infancy and influence the further development of a child affected by GDM.

## 1. Introduction

Gestational diabetes mellitus (GDM) is one of the most common complications during pregnancy. Both gestational diabetes and pregestational diabetes are responsible for several complications in the perinatal and neonatal periods. Therefore, early diagnosis of GDM and early treatment are the main elements of prevention of complications such as birth defects [1], macrosomia [2], perinatal injuries [3], respiratory distress syndrome [4], transient respiratory distress [5], cardiomyopathy [6], hyperbilirubinemia [7], and metabolic disorders [8].

The troponin complex consists of three subunits, troponin C (TnC), troponin I (TnI), and troponin T (TnT). In cardiac and skeletal muscles, troponin C binds calcium that coordinates the Ca2 + -regulatory system of the muscle, troponin T controls interactions between actin and myosin, and troponin I exerts inhibitory properties. Troponins I and T have been found mainly in cardiac muscles, whereas Troponin C is present in cardiac and skeletal muscles [9]. Cardiac TnT of molecular weight 39.7 kDa differs from skeletal muscle TnT by its isoform content. However, the cardiac isoform of TnT is expressed only in cardiac muscles. Thus, cardiac troponin T (cTnT) is a highly sensitive cardio-specific marker of myocardial damage. In response to myocardial ischemia, cardiac troponin is released into the bloodstream and can be used to diagnose myocardial infarction. Troponin T is present in the peripheral blood of healthy newborns [10]. Two studies have reported a clinical relationship between troponin peripheral blood levels and the clinical status of a newborn [11]. TnT in the cord blood of neonates has been shown to be independent of gestation weight and sex [12]. However, only a few studies reported relationships between TnT levels in peripheral blood in neonates [13]. In this study, we compare TnT levels in the peripheral blood of healthy newborns and newborns from diabetic mothers to verify the usefulness of high-sensitivity troponin T (hs-TnT) in predicting possible complications caused by GDM.

## 2. Patients and Methods

### 2.1. Ethics

The study was carried out according to the Declaration of Helsinki of the World Medical Association (WMA) [14]. The study was approved by the Ethics Committee of the Kazimierz Pulaski University of Technology and Humanities: KB/154/2009. Parents of the examined children provided a signed informed consent form to participate in the study.

### 2.2. Study Participants

In the study, we used data from blood examinations of newborns whose mothers were hospitalized in the Department of Neonatology, Department of Obstetrics, Feminine Diseases, Gynecology Oncology, and Regional Hospital Bródnowski, Warsaw, Poland. A total of *N* = 86 newborns were studied and stratified into two groups: 52 healthy (26 male and 26 female), that is, newborns with body weight ≥ 10th percentile, born in good condition (APG 8-10pts) from pregnancy not complicated by diabetes, and 34 (17 male and 17 female) born to mothers with type 1 or type 2 diabetes. The statistical description of the study group is shown in Table 1.

### 2.3. Experimental Methods

Heparinized plasma of blood from the neonates was collected by venepuncture in a tube with lithium and heparin. The collected samples were chilled to 4°C and centrifuged at 1500 rpm, and the serum was collected. The serum collected was stored at -20°C until laboratory tests were performed.

The concentration of cardiac troponin T hs was measured using the Cobas Elecsys assay in neonatal blood serum on a Cobas 8000 analyzer. The assay consisted of two steps: in step 1, a complex was incubated, for which a biotinylated antigen was used with a monoclonal antibody specific for cardiac troponin T, and a monoclonal antibody specific for cardiac troponin T that was labeled with a ruthenium complex. In step 2, incubation with particles coated with streptavidin. In this step, the complex is bound to the solid phase as a result of the affinity of biotin and streptavidin.

The mixture was then transferred to a measuring chamber in which the tropomyosin T concentration was measured by the electrochemiluminescence level. The tropomyosin concentration was expressed in ng/mL.

### 2.4. Statistical Analysis

All statistical analyses were performed using the R package [15]. The normality of a sample distribution was assessed using the Shapiro-Wilk normality test. The distributions of means and differences in means between survivors and deceased patients were tested using a bootstrap test consisting of 10,000 repeats with replacement.

## 3. Results

Since there is a lack of statistical differences in studied parameters between the genders and neonates, the results are analyzed only as a function of the experimental group, that is, GDM—children born from diabetic mothers and neonates born from healthy mothers. The statistical description of the groups studied is shown in Table 2.

**Figure 1:**
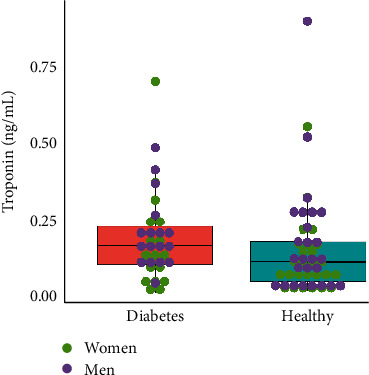
Box-plot representation of troponin levels stratified by study group. Healthy, 52 healthy (26 male and 26 female), that is, newborns with bodyweight ≥ 10th percentile, born in good condition (APG 8-10pts) from pregnancy not complicated by diabetes and GDM, 34 (17 male and 17 female) born to mothers with type 1 or type 2 diabetes.

The box-plot representation of troponin levels stratified by study group is shown in Figure 1. Table 1 revealed similar minimal troponin levels in GDM and healthy groups: 0.02 ng/mL and 0.028 ng/mL, respectively. The healthy group is defined by the higher maximal troponin level (0.895 ng/mL) than this observed in the GDM group (0.701 ng/mL). The upper limit interval for GDM (0.524 ng/mL) is analogous to the healthy group (0.546 ng/mL).

## 4. Discussion

Analysis of the current literature indicates that an increase in cardiac troponin T and I (cTnT and cTnI) is not limited only to myocardial infarction [16]. For example, high-sensitivity cardiac troponin T (hs-cTnT) is associated with the incidence of diabetes [17] and acute ischemic stroke [18]. Several reports studied relations between blood cTnT levels and neonates' health status [19–24]. As shown in previous studies [25, 26], the size of troponin molecules prevents crossing the placenta, and therefore mother blood levels of cTnT cannot influence neonatal serum troponin levels. Therefore, the observed differences between the study groups are only caused by postpartum physiological differences between newborns. This study showed that the first, median, and third quartile of serum troponin levels in GDM are greater than in the healthy group. Since gestational diabetes mellitus can cause multiple complications in newborns, including macrosomia [27], hypocalcemia [28], hypertrophic cardiomyopathy [29], and cardiac malformations [30], it may also be responsible for the observed increase in the serum troponin levels in neonates from diabetic mothers. However, more studies are required to confirm or disprove this observation.

There is a difference between the median troponin levels in healthy neonates reported in this study (0.12 ng/mL) and those reported by Trevisanuto et al. [24] (0.17 ng/mL). Furthermore, the median serum troponin levels of the GDM (0.171 ng/mL) is virtually equal to the values reported for a healthy group by Trevisanuto et al. [24].

However, the results of this study confirmed the previous study that indicated that hs-cTnT levels do not conform to a Gaussian distribution in children older than two days [13]. Furthermore, this study confirms the upper limit interval of troponin levels for ages between 2 and 28 days [13]. Furhtermore, the previous study reported that the upper limit of troponin levels in healthy children was equal to 0.608 ng/mL, while this study reported 0.546 ng/mL.

## 5. Conclusions

Identifying marker that allow for the prediction of the health of newborns is of paramount importance from a medical point of view. This study tested the hypothesis of the potential diagnostic applicability of serum troponin levels in neonates. The observed differences, although statistically insignificant, between healthy newborns and those born to diabetic mothers can be indicative of possible health problems that can appear during childhood and may influence the future development of a child. However, follow-up studies are required to verify this hypothesis.

## Figures and Tables

**Table 1:** Statistical description of study groups: GDM, born to mothers with type 1 or type 2 diabetes; healthy newborns with bodyweight ≥ 10th percentile, born in good condition (APG 8-10pts) from pregnancy not complicated by diabetes.

Group	*n*	Variable	Min	Max	Median	Mean	Sd
GDM	34	Neonate age (hr)	24	120	72	72	24.361
		Mother age	25	41	31	31.941	4.376
		Pregnancy	1	4	1	1.647	0.849

Healthy	52	Neonate age (hr)	0	120	72	64.154	24.104
		Mother age	20	39	30.5	29.962	4.593
		Pregnancy	1	4	2	1.846	0.916

**Table 2:** The statistical description of blood serum troponin levels in GDM born to mothers with type 1 or type 2 diabetes and healthy newborns with bodyweight ≥ 10th percentile, born in good condition (APG 8-10pts) from pregnancy not complicated by diabetes.

Group	Variable	*n*	Min (ng/mL)	Max (ng/mL)	Median (ng/mL)	Mean (ng/mL)	Sd (ng/mL)	97.5th percentiles (ng/mL)
1	GDM	34	0.02	0.701	0.171	0.197	0.142	0.524
2	Healthy	52	0.028	0.895	0.12	0.158	0.155	0.546

## Data Availability

The data reported in this study are available on request from the authors.
